# Editorial Department

**Published:** 1855-03

**Authors:** 


At a meeting of the Medical Class of the University of Buffalo, at the close of Professor Hunt's Course of Anatomy, the following resolutions were unanimously adopted:

*Resolved*, That we, the students of the class of '55, do most heartily recognize, and will always gratefully remember, the untiring care for our advancement, and solicitude for our success, manifested towards us by Prof. Hunt, both as teacher and friend.

*Resolved*, That we do sincerely congratulate him upon his successful debut as a teacher in one of the deepest of the circle of the sciences, and that we do earnestly hope that he may long remain its successful and eloquent expositor.

*Resolved*, That though about to separate from him, he may always be assured of our love for him, and of our gratitude for the services he has rendered us.

*Resolved*, That the above resolutions be transmitted to Professor Hunt, as the spontaneous expression of the unanimous sentiment of the class.

